# Cerebellar Cognitive Affective Syndrome in Spinocerebellar Ataxia Type 6

**DOI:** 10.1007/s12311-026-02056-5

**Published:** 2026-08-12

**Authors:** Fernanda Mary Machado, Breno Kazuo Massuyama, July Silveira Gomes, Marcondes Cavalcante França, Orlando Graziani Povoas Barsottini, José Luiz Pedroso

**Affiliations:** 1 Division of General Neurology and Ataxia Unit, Department of Neurology and Neurosurgery, Federal University of São Paulo (UNIFESP), São Paulo, Brazil; 2 Schizophrenia Program, Department of Psychiatry, Federal University of São Paulo (UNIFESP), São Paulo, Brazil; 3 Albert Einstein Israeli Faculty of Health Sciences, São Paulo, Brazil; 4 Department of Neurology, School of Medical Sciences, University of Campinas (UNICAMP), Campinas, Brazil; 5 Department of Neurology, Federal University of Sao Paulo, 650 Pedro de Toledo Street, Sao Paulo, Brazil

**Keywords:** Cerebellar cognitive affective syndrome, Spinocerebellar ataxia, Spinocerebellar ataxia type 6, Cognition, Executive function, Neuropsychological tests

## Abstract

Background: Spinocerebellar ataxia type 6 (SCA6) has traditionally been classified as a "pure" cerebellar syndrome; however, converging evidence points to non-motor involvement. Characterization of its cognitive-affective profile remains limited, constraining the understanding of its full clinical spectrum. Objectives: To investigate non-motor manifestations in SCA6 and contribute to a more comprehensive characterization of its cognitive-affective profile through standardized neuropsychological assessment. Methods: A monocentric, cross-sectional case-control study included nine patients with genetically confirmed SCA6 and eight healthy controls matched for age and years of education. Participants were assessed using the Cerebellar Cognitive Affective/Schmahmann Syndrome Scale (CCAS-S), the Scale for the Assessment and Rating of Ataxia (SARA), and a comprehensive neuropsychological battery. Anxiety and depressive symptoms were also assessed. Results: Compared with controls, SCA6 patients showed poorer performance on time-dependent measures sensitive to inhibitory control and selective attention and on tasks involving visuoconstructive and visuomotor integration, as well as poorer verbal episodic memory, with a trend toward poorer confrontation naming; motor slowing and reduced processing speed cannot be ruled out. Anxiety was significantly elevated. Cerebellar motor dysfunction predicted a higher number of failed CCAS-S domains, even after controlling for disease duration. Conclusions: These exploratory findings suggest cognitive and affective alterations in SCA6 beyond the motor domain, warranting investigation within the Cerebellar Cognitive Affective Syndrome framework. Given the small sample, absence of correction for multiple comparisons, and potential motor and processing-speed confounds, results are preliminary. They highlight the relevance of targeted neuropsychological assessment and may inform more individualized care strategies.

## Introduction

Spinocerebellar ataxias (SCAs) are considered autosomal dominant neurodegenerative disorders that affect the cerebellum and its connections [1]. Among them, Spinocerebellar ataxia type 6 (SCA6) is a late-onset autosomal dominant neurodegenerative disorder caused by a CAG repeat expansion in the CACNA1A gene, which encodes the α1A subunit of P/Q-type calcium channels [2]. Neuropathology shows selective Purkinje cell loss and secondary cerebellar cortical atrophy, with relative preservation of other brain regions [3]. Following Purkinje cells degeneration over the years, the symptoms become progressively more evident. Balance difficulties often appear first and worsen with disease progression [4]. This cerebellar focus defines SCA6 as an isolated syndrome.

However, growing evidence suggests that extracerebellar and non-motor dysfunctions extend beyond the cerebellum itself [5]. Cognitive alterations across domains, particularly executive and attentional deficits, have been reported, with reduced IQ observed in most SCAs except SCA3 and lower verbal intelligence quotient (IQ) in SCA1 and SCA6 [6, 7]. The cerebellar cognitive affective syndrome (CCAS), described by Schmahmann and Sherman, involves deficits in executive, linguistic, visuospatial, and affective regulation, reflecting a “dysmetria of thought” analogous to motor ataxia [8]. Recent evidence shows that SCA includes not only motor and vestibular syndromes but also CCAS related cognitive and neuropsychiatric features [9]. Selvadurai et al. applied the Cerebellar Cognitive Affective/Schmahmann Syndrome Scale (CCAS-S), a brief bedside tool developed to screen for and quantify CCAS, in a large cohort including several SCA subtypes and found reduced CCAS-S performance in SCA2, SCA3 and SCA6 compared with controls, without clearly distinct subtype-specific patterns [10]. In hereditary ataxias, Thieme et al. showed that the CCAS-Scale is useful to detect cognitive involvement at the group level, particularly in SCA3, but that substantial overlap between mildly affected patients and controls leads to many false positives and blunts differences between subtypes. This limitation highlights the need for more refined neuropsychological protocols to define the subtype specific cognitive affective profile of ataxias [11].

Following this premise, this study examined cognitive and affective alterations in individuals with genetically confirmed SCA6 using the CCAS-S and a comprehensive neuropsychological battery, with the specific aim of helping to fill current gaps in the literature regarding the delineation of the subtype-specific cognitive-affective profile of this disorder. Moreover, the study intended to establish correlations between the number of failed CCAS-S domains, neuropsychological performance, and clinical measures of motor and affective dysfunction, in order to contribute to a more precise delineation of the cognitive-affective profile of SCA6 and to provide a new perspective on its classification as a ‘pure’ cerebellar ataxia.

## Methods

### Study Design

We conducted a monocentric, cross-sectional, case–control study. To assemble the clinical group, we selected patients with genetically confirmed SCA6 who were under longitudinal follow-up at the Neurology and Ataxia Department of the Federal University of São Paulo, the same institution in which the study was conducted, between 2022 and 2025. Whereas control participants were gathered through active invitations within the researcher’s professional networks. The final sample comprised nine SCA6 patients and eight healthy controls matched for age and education. Controls met criteria of age ≥ 40, education ≥ 16 years, and estimated IQ ≥ 70. Exclusion criteria comprised neurological or psychiatric disorders, substance abuse, or asymptomatic carrier status. On average, the individualized administration of the neuropsychological assessment instruments required approximately two and a half hours. All participants provided written informed consent. The study was approved by the UNIFESP Ethics Committee (approval no. 5.908.933; CAAE 65187722.0.0000.5505) in accordance with the Declaration of Helsinki.

### Variables

The primary outcome variable was the number of failed domains on the CCAS-S. Secondary outcome variables comprised performance scores on each neuropsychological test. Independent variables included group status (SCA6 vs. controls), age, years of education, and estimated IQ. Clinical variables were the SARA total score and disease duration. Affective variables corresponded to the total scores on the HAM-A and BDI-II.

### Data Collection: Clinical and Neuropsychological Assessment

The *Cerebellar Cognitive Affective/Schmahmann Syndrome Scale* (CCAS-S) was the primary instrument, applied in its Brazilian Portuguese version, translated, culturally adapted, and validated by Scott et al. [12]. Originally developed by Hoche et al., the scale comprises ten domains assessing semantic and phonemic verbal fluency, category switching, forward and backward digit span, cube copying, verbal recall, abstraction and similarities, go/no-go performance, and affective symptoms. Each domain yields a raw score, and the total score (0–120) reflects overall cognitive-affective functioning [13]. A categorical diagnostic system classifies zero failed domains as normal, one as possible CCAS, two as probable, and three or more as definite CCAS. The CCAS-S has been validated in several languages and shown sensitivity to detect cognitive-affective deficits in SCAs, particularly in verbal fluency [14].

In addition, a comprehensive neuropsychological battery was administered, including the *Wechsler Abbreviated Scale of Intelligence* (WASI) to estimate global intellectual functioning (IQ); the *Boston Naming Test* (BNT) for confrontation naming; the *Rey Auditory Verbal Learning Test* (RAVLT) for verbal learning and memory; the *Rey–Osterrieth Complex Figure Test* (ROCF) for visuoconstructive abilities and visuospatial memory; the *Stroop Color and Word Test* (Stroop Test) for selective attention; the *Corsi Block-Tapping Test* (Corsi Blocks) for visuospatial working memory and immediate span, the *Hayling Sentence Completion Test* (Hayling Test) for executive functioning and the *Tower of London* (ToL) for planning and executive control; and the *Hamilton Anxiety Rating Scale* (HAM-A) together with the *Beck Depression Inventory–II* (BDI-II) for affective symptoms. We also included the Picture Completion subtest from the *Wechsler Adult Intelligence Scale–Third Edition* (WAIS-III) to assess visual perception and attention to detail. Exploratory correlations examined associations between the number of failed CCAS-S domains and neuropsychological performance, as well as the relationship between the number of failed CCAS-S domains and the *Scale for the Assessment and Rating of Ataxia* (SARA) as an index of disease severity. All instruments were administered in their standardized Brazilian-Portuguese versions, following normative procedures and scoring criteria established for the local population [15–26].

### Statistical Analysis

#### Group Comparisons

Group differences between SCA6 patients and controls were examined for the number of failed CCAS-S domains and for sociodemographic, clinical, and neuropsychological variables. Given the small sample size and the exploratory nature of the study, between-group comparisons were performed using independent samples Student’s t-tests (two-tailed) without prior formal testing of distributional assumptions; results should therefore be interpreted as preliminary. Effect sizes were calculated using Cohen’s d [27], and 95% confidence intervals for mean differences were computed using Welch’s correction for unequal variances [28].

#### Descriptive Statistics

Descriptive statistics (means, medians, standard deviations, and standard errors) were calculated for all variables. For the CCAS-S, analyses focused on the total number of failed domains, which were compared between groups.

#### Correlation Analyses

Exploratory correlations in the SCA6 group examined relationships between the number of failed CCAS-S domains, SARA scores, and neuropsychological performance, as well as between cognitive, motor, and affective measures (BDI-II, HAM-A). Distributional normality of each variable in the SCA6 group was assessed using the Shapiro–Wilk test. Pearson correlation coefficients were applied to variables meeting the normality assumption; Spearman’s rho was used for the Stroop Test and the Rey–Osterrieth Complex Figure copy, which did not meet the normality criterion (Shapiro–Wilk, *p* < 0.05). 95% confidence intervals for correlation coefficients were computed using Fisher’s z transformation [29].

#### Software and Significance

All statistical analyses were conducted in R (version 4.4.1; RStudio) [30], with figures generated using ggplot2 [31]. Additionally, a single correlation analysis was performed in Python 3.12.10, using the SciPy, NumPy, and pandas libraries; the corresponding visualization was produced with Matplotlib and seaborn [32–37]. All tests were two-tailed, and statistical significance was set at *p* < 0.05. Given the exploratory nature of the study and the small sample size, no correction for multiple comparisons was applied; findings should therefore be interpreted with caution, as the risk of both false-positive and false-negative results is elevated.

## Results

### Demographic and Clinical Characteristics

The sample included 9 individuals with SCA6 and 8 healthy controls. Overall, no significant differences were found between groups regarding age or years of education, whereas estimated IQ showed a borderline difference (*p* = 0.051), with lower scores in the SCA6 group; the corresponding data are presented in Table 1. Within the SCA6 group, exploratory Spearman correlations indicated that the number of failed CCAS-S domains was not significantly associated with age (ρ = 0.383, *p* = 0.309; 95% CI [− 0.377, 0.835]) or years of education (ρ = −0.433, *p* = 0.244; 95% CI [− 0.852, 0.324]), suggesting that these demographic variables did not substantially influence the number of failed CCAS-S domains.

**Table 1:** Demographic and global measures for the SCA6 and control groups

	Controls (*n* = 8)	SCA6 (*n* = 9)	Mean difference (95% CI)	Cohen’s d	*p*-value
Age (years)	63.6 ± 10.9 (SE 3.8)	64.9 ± 11.3 (SE 3.8)	1.26 [− 10.22, 12.74]	0.11	0.818
Years of education	18.3 ± 2.6 (SE 0.9)	16.8 ± 1.9 (SE 0.6)	−1.47 [− 3.84, 0.90]	−0.67	0.190
Estimated IQ	117.3 ± 15.7 (SE 5.6)	99.4 ± 18.5 (SE 6.2)	−17.81 [− 35.50, − 0.11]	−1.03	0.051

Note. Values are mean ± SD; standard error (SE) in parentheses. Mean difference is expressed as SCA6 minus Controls. 95% confidence intervals (CI) were calculated using Welch’s correction for unequal variances. Cohen’s *d* reflects effect size magnitude: small (0.2), medium (0.5), large (0.8). Group comparisons were performed using independent samples Student’s *t*-test (two-tailed). Statistical significance was set at *p* < 0.05. No correction for multiple comparisons was applied. Given the small sample size and the absence of correction for multiple comparisons, results should be interpreted as exploratory

### Executive Functions

Performance on the Hayling Test, measured by total completion time (mean ± SD, seconds), a time-dependent measure sensitive to inhibitory control, was poorer in the SCA6 group (117.60 ± 74.50, SE 26.34) than in controls (45.29 ± 17.67, SE 6.25; mean difference 72.31, 95% CI [9.58, 135.03]; *p* = 0.01; d = 1.34). Performance on the Stroop Test, assessed by completion time (mean ± SD, seconds), a time-dependent measure sensitive to selective attention, was also poorer in patients (19.82 ± 4.12, SE 1.37) than in controls (14.30 ± 2.95, SE 1.04; mean difference 5.52, 95% CI [1.83, 9.21]; *p* = 0.007; d = 1.52). The contribution of motor slowing, reduced processing speed, and verbal output demands to performance on these time-dependent measures cannot be ruled out. In contrast, cognitive flexibility on the Hayling Test (mean ± SD; SCA6: 5.91 ± 3.05, SE 1.08; controls: 4.00 ± 1.38, SE 0.49; mean difference 1.90, 95% CI [− 0.74, 4.54]; *p* = 0.13; d = 0.80) and executive planning on the Tower of London (total score, mean ± SD; SCA6: 32.80 ± 1.79, SE 0.80; controls: 28.00 ± 7.12, SE 2.69; mean difference 4.80, 95% CI [− 1.83, 11.43]; *p* = 0.17; d = 0.85) did not differ significantly between groups.

### Memory

Immediate visuospatial memory, assessed with the Corsi Blocks Forward (maximum span, mean ± SD), was slightly lower in the SCA6 group (7.67 ± 2.29, SE 0.76) than in controls (9.00 ± 1.60, SE 0.57; mean difference − 1.33, 95% CI [− 3.37, 0.70]; *p* = 0.190; d = − 0.67), although this difference was not statistically significant. Visuospatial working memory, measured with the Corsi Blocks Backward (maximum span, mean ± SD), followed a similar trend (SCA6: 6.67 ± 1.94, SE 0.65; controls: 8.38 ± 2.62, SE 0.93; mean difference − 1.71, 95% CI [− 4.15, 0.73]; *p* = 0.144; d = − 0.75), without reaching statistical significance. Verbal episodic memory, evaluated with the RAVLT delayed recall score (mean ± SD), was significantly reduced in SCA6 patients (6.44 ± 2.40, SE 0.80) compared with controls (12.00 ± 2.39, SE 0.85; mean difference − 5.56, 95% CI [− 8.04, − 3.07]; *p* < 0.001; d = − 2.32). Visuoconstructive and visual episodic memory abilities, assessed with the Rey–Osterrieth Complex Figure (copy and delayed recall scores, mean ± SD; maximum = 36 points), a task involving visuoconstructive and visuomotor integration, graphomotor skills, and organizational planning, showed trends toward poorer performance in the SCA6 group both on the copy condition (SCA6: 29.83 ± 8.63, SE 2.88; controls: 36.00 ± 0.00, SE 0.00; mean difference − 6.17, 95% CI [− 12.80, 0.47]; *p* = 0.062; d = − 0.98) and delayed recall (SCA6: 14.28 ± 8.95, SE 2.98; controls: 20.81 ± 6.39, SE 2.26; mean difference − 6.53, 95% CI [− 14.54, 1.47]; *p* = 0.108; d = − 0.83), although these differences did not reach statistical significance. The contribution of motor and visuomotor factors to performance on this task cannot be ruled out.

### Language - Naming Abilities

Within the language domain, confrontation naming, assessed with the Boston Naming Test (total correct responses, mean ± SD; maximum score = 60), showed a trend toward poorer performance in the SCA6 group (52.11 ± 6.47, SE 2.16) compared with controls (56.50 ± 2.78, SE 0.98; mean difference − 4.39, 95% CI [− 9.60, 0.82]; *p* = 0.096; d = − 0.86), although this difference was not statistically significant.

### Logical Reasoning and Visuoconstructive Skills

Performance on the WASI Block Design subtest (total raw score, mean ± SD), a task involving visuoconstructive and visuomotor integration, constructional praxis, and manual dexterity under timed administration, was poorer in patients with SCA6 (27.4 ± 13.42, SE 4.74) than in controls (46.4 ± 9.93, SE 3.51; mean difference − 19.00, 95% CI [− 31.76, − 6.24]; *p* = 0.006; d = − 1.61). The contribution of motor and visuomotor factors to this difference cannot be ruled out. In contrast, performance on WASI Matrix Reasoning (total raw score, mean ± SD; SCA6: 21.9 ± 7.70, SE 2.57; controls: 26.5 ± 4.81, SE 1.70; mean difference − 4.61, 95% CI [− 11.24, 2.02]; *p* = 0.16; d = − 0.71) did not differ significantly between groups.

### Emotional-Affective Symptoms and CCAS-S

**Fig. 1:**
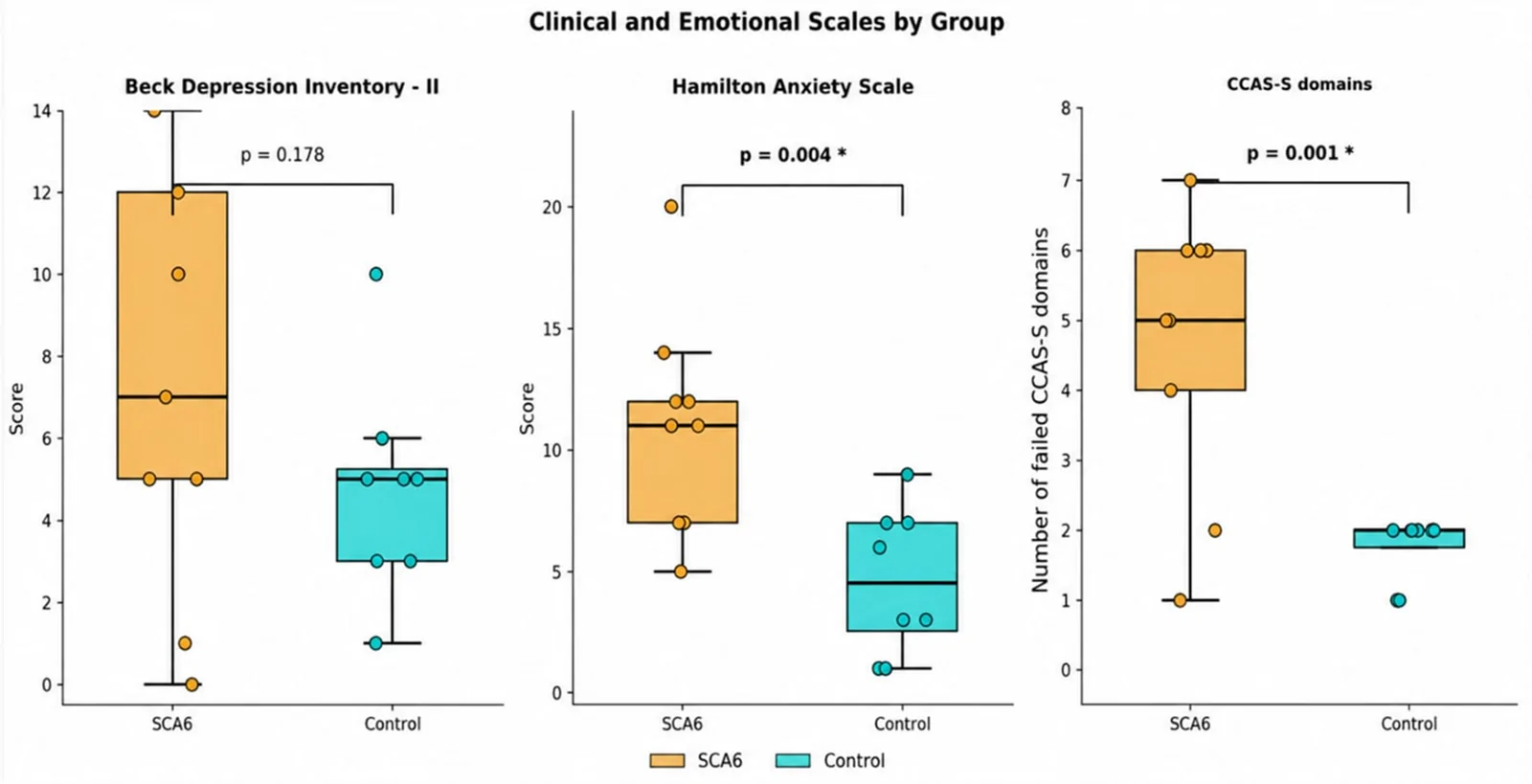
Clinical and emotional scales by group. Note. Boxplots display median, interquartile range, and individual data points for the Beck Depression Inventory-II (BDI-II), Hamilton Anxiety Scale (HAM-A), and the number of failed CCAS-S domains. Orange boxes represent the SCA6 group and cyan boxes represent the control group. Asterisks indicate statistically significant between-group differences (* p < 0.05)

Anxiety symptoms, assessed with the HAM-A, were significantly higher in the SCA6 group (mean 11.00 ± 4.47, SE 1.49) than in controls (mean 4.63 ± 3.02, SE 1.07; mean difference 6.38, 95% CI [2.44, 10.31]; *p* = 0.004; d = 1.65). For reference, the conventional HAM-A severity thresholds are: 0–7, no anxiety; 8–14, mild anxiety; 15–23, moderate anxiety; ≥24, severe anxiety. Depressive symptoms, assessed with the BDI-II, were higher in the SCA6 group (mean 8.56 ± 7.16, SE 2.39) than in controls (mean 4.75 ± 2.66, SE 0.94; mean difference 3.81, 95% CI [− 1.88, 9.49]; *p* = 0.178; d = 0.69), although this difference did not reach statistical significance. The number of failed CCAS-S domains was higher in the SCA6 group (mean 4.67 ± 2.00, SE 0.67) compared with controls (mean 1.75 ± 0.46, SE 0.16; mean difference 2.92, 95% CI [1.36, 4.47]; *p* = 0.001; d = 1.95), with a higher number of failed domains and a shift toward more severe categories. Figure 1 illustrates these results.

In accordance with the original CCAS-S criteria, no control participant met criteria for definite CCAS. Among SCA6 patients, 77.8% were classified as definite CCAS, 11.1% as probable, and 11.1% as possible. In the control group, 75.0% were classified as probable and 25.0% as possible CCAS. Table 2 presents the CCAS-S classification in each group.

**Table 2:** CCAS-S classification (definite, probable, possible) in SCA6 and control groups

Classification	Controls (*n* = 8)		SCA6 (*n* = 9)	
	*N*	%	*N*	%
Possible	2	25.0	1	11.1
Probable	6	75.0	1	11.1
Definite	0	0.0	7	77.8

Note. Classification according to the original CCAS-S criteria (Hoche et al., 2018): possible CCAS = one failed domain; probable CCAS = two failed domains; definite CCAS = three or more failed domains. Categories should be interpreted with caution given the small sample size and the limited specificity of the possible and probable categories

### Correlation Analyses

In the SCA6 group, exploratory correlation analyses revealed significant associations between the number of failed CCAS-S domains and performance on the Boston Naming Test (*r* = − 0.721, *p* = 0.028; 95% CI [− 0.937, − 0.110]), Block Design (*r* = − 0.758, *p* = 0.029; 95% CI [− 0.953, − 0.114]), estimated IQ (*r* = − 0.698, *p* = 0.037; 95% CI [− 0.931, − 0.062]), Stroop Test (ρ = 0.758, *p* = 0.018; 95% CI [0.188, 0.946]), and Rey Figure Copy (ρ = −0.704, *p* = 0.034; 95% CI [− 0.932, − 0.076]).

**Fig. 2:**
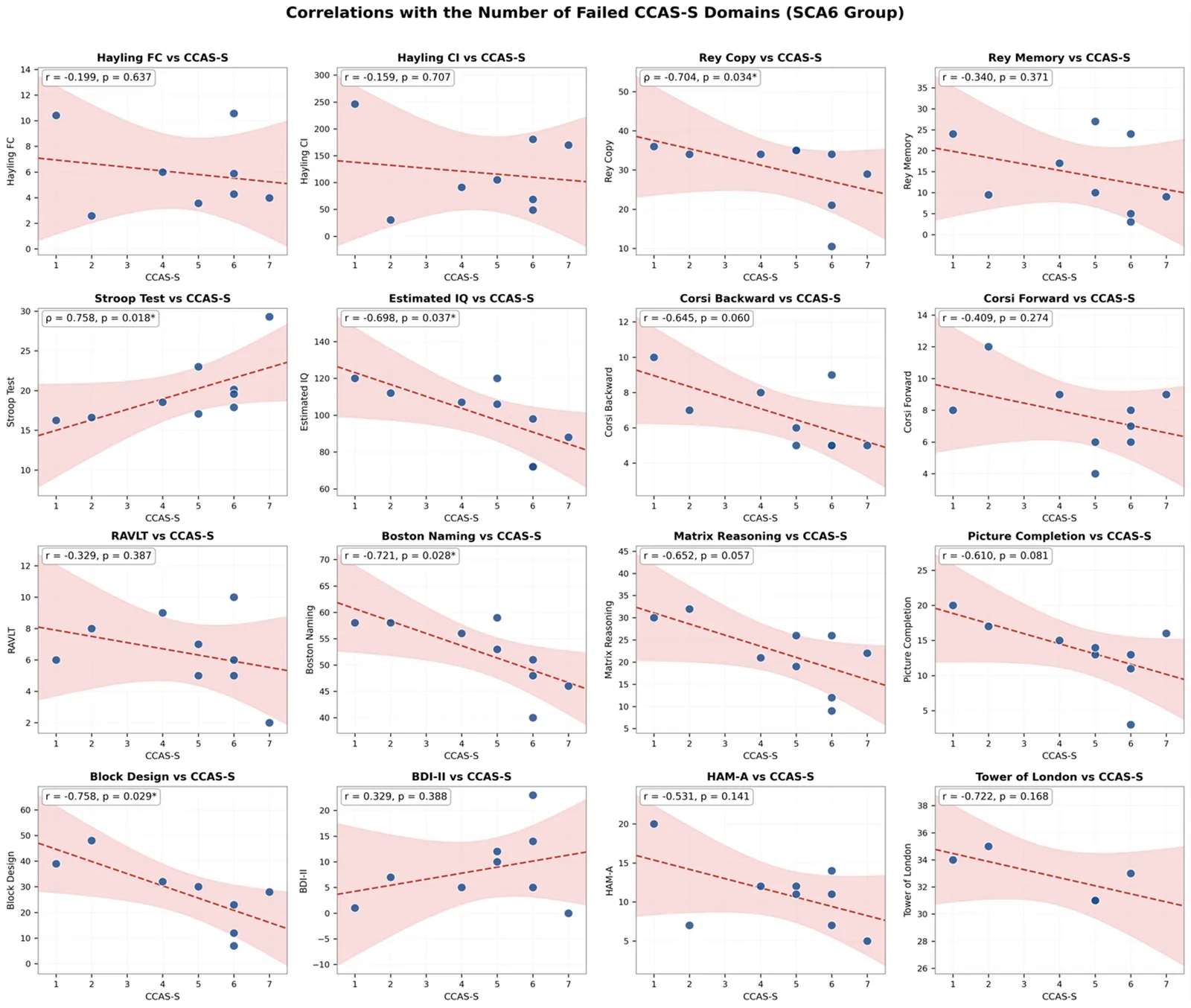
Correlations between CCAS-S and cognitive performance in SCA6. Note. Scatterplots showing relationships between the number of failed CCAS-S domains and neuropsychological measures in the SCA6 group (n = 9 for most analyses; n = 8 for Block Design, due to missing data). Pearson correlation coefficients (r) were applied for variables meeting normality assumptions (Shapiro–Wilk p > 0.05); Spearman's ρ was used for Stroop Test and Rey Figure Copy, which did not meet normality criteria. Dashed lines represent linear fit with 95% confidence intervals. Asterisks indicate statistically significant correlations (* p < 0.05). All analyses are exploratory

Additional non-significant trends were observed for Corsi Blocks Backward (*r* = − 0.645, *p* = 0.060; 95% CI [− 0.917, 0.033]), Matrix Reasoning (*r* = − 0.652, *p* = 0.057; 95% CI [− 0.918, 0.022]), Picture Completion (*r* = − 0.610, *p* = 0.081; 95% CI [− 0.907, 0.091]), Rey Figure Memory (*r* = − 0.340, *p* = 0.371; 95% CI [− 0.819, 0.419]), RAVLT (*r* = − 0.329, *p* = 0.387; 95% CI [− 0.815, 0.428]), and HAM-A (*r* = − 0.531, *p* = 0.141; 95% CI [− 0.884, 0.206]). No relevant associations were identified for Hayling Test, cognitive flexibility (*r* = − 0.199, *p* = 0.637; 95% CI [− 0.792, 0.588]), inhibitory control (*r* = − 0.159, *p* = 0.707; 95% CI [− 0.777, 0.615]), Corsi Blocks Forward (*r* = − 0.409, *p* = 0.274; 95% CI [− 0.844, 0.350]), BDI-II (*r* = 0.329, *p* = 0.388; 95% CI [− 0.429, 0.815]), or Tower of London (*r* = − 0.722, *p* = 0.168; 95% CI [− 0.980, 0.442]). Given the small sample size (*n* = 9 for most analyses; *n* = 8 for Block Design, due to missing data), all estimates are sensitive to individual observations and should be interpreted with caution (Fig. 2).

**Fig. 3:**
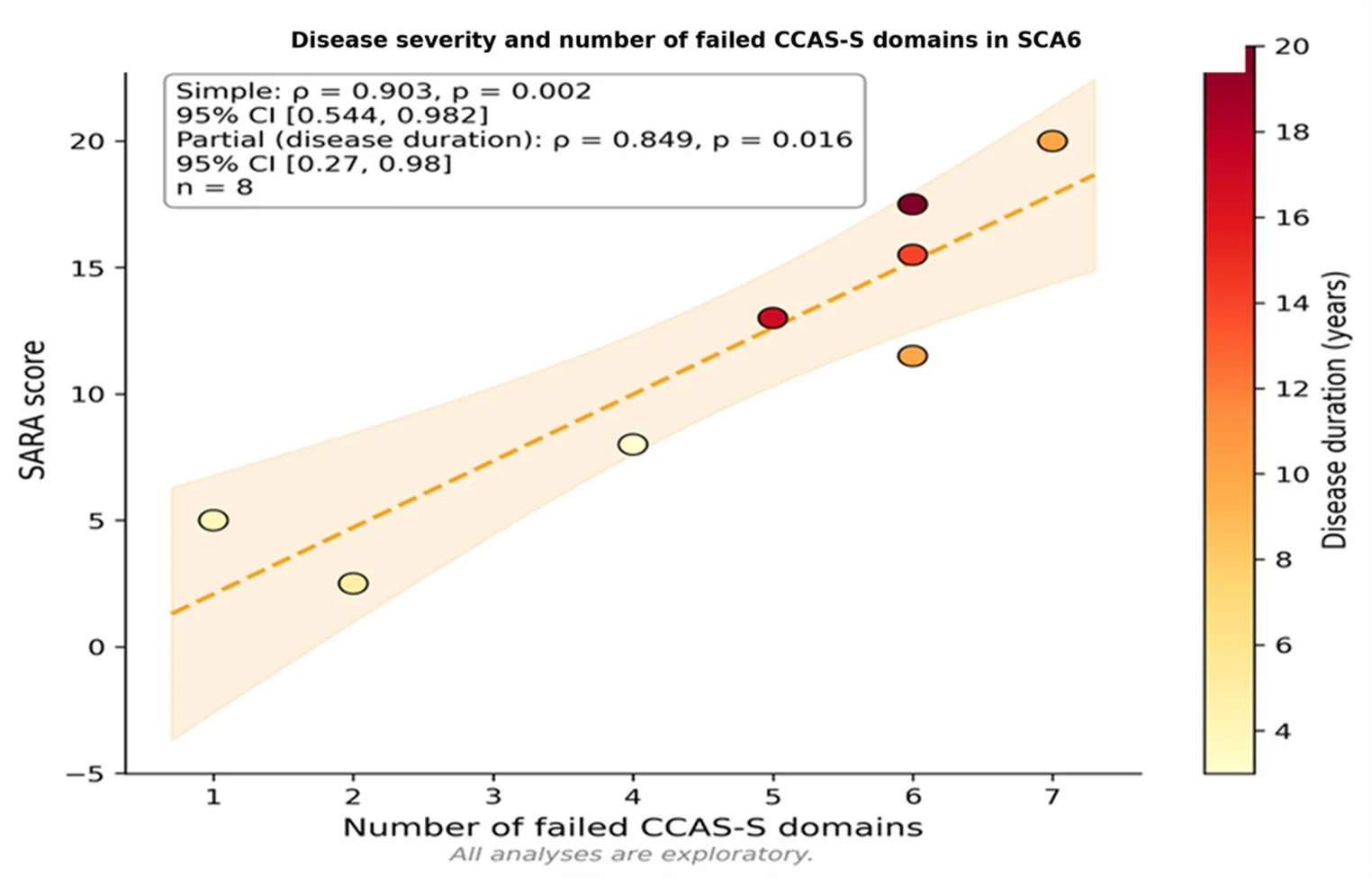
Disease severity and the number of failed CCAS-S domains in SCA6. Note. Scatterplot showing the relationship between SARA scores and the number of failed CCAS-S domains in SCA6 patients (n = 8). Each point represents an individual participant, colored by disease duration. Dashed line represents linear fit with 95% confidence interval (shaded area). Simple and partial correlations were computed using Spearman's ρ. Simple correlation: ρ = 0.903, p = 0.002; 95% CI [0.544, 0.982]. Partial correlation controlling for disease duration: ρ = 0.849, p = 0.016; 95% CI [0.27, 0.98]. All analyses are exploratory

Both simple and partial correlations were computed between SARA scores and the number of failed CCAS-S domains. The simple correlation yielded ρ = 0.903 (*p* = 0.002; 95% CI [0.544, 0.982]; *n* = 8), indicating that greater motor severity was associated with a higher number of failed CCAS-S domains. The partial correlation controlling for disease duration remained high and statistically significant (ρ = 0.849, *p* = 0.016; 95% CI [0.27, 0.98]), suggesting that the association between motor severity and cognitive-affective impairment persists independently of disease duration. Figure 3 graphically illustrates the exploratory correlation results.

## Discussion

This study examined cognitive and affective functions in SCA6 through a comprehensive neuropsychological assessment. SCA6 patients showed poorer performance on time-dependent measures sensitive to inhibitory control and selective attention, on tasks involving visuoconstructive and visuomotor integration, on verbal episodic memory and on language measures, along with higher levels of anxiety. The combination of the CCAS-S and the neuropsychological battery was sensitive to non-motor alterations and correlated with SARA scores, consistent with the possibility that motor and non-motor manifestations in SCA6 may share common cerebellar substrates.

Previous studies suggest that patients with spinocerebellar ataxias may exhibit cognitive and affective alterations even in early stages of the disease, a pattern that has been described under the framework of CCAS and is screened by the CCAS-S [38]. In our sample, SCA6 patients showed a lower estimated IQ than controls. In the absence of premorbid data, and considering that the number of failed CCAS-S domains was not significantly associated with age or years of education in this sample, the results are consistent with the possibility of intellectual decline related to disease progression, in line with previous reports of scores approximately one standard deviation below the mean [39]. Reductions in verbal IQ have also been described in SCA1 and SCA6 [7]. In our study, the most pronounced impairments were observed in fluid intelligence, with a borderline difference between groups (*p* = 0.051). Although these limitations restrict more precise interpretations, it cannot be ruled out that the high educational level of the sample may have attenuated part of these deficits.

In the executive domain, our patients showed longer completion times on the Hayling and Stroop tests, time-dependent measures sensitive to inhibitory control and selective attention. It should be noted, however, that time-based performance measures are susceptible to alterations arising from motor impairments caused by cerebellar lesions, a methodological challenge previously highlighted in longitudinal neuropsychological studies of hereditary ataxias [40]. The extent to which these factors contributed to the observed results cannot be determined from the available data. Nonetheless, the findings may reflect difficulties in behavioral self-regulation and in managing competing environmental demands, consistent with previous reports of impairment in tasks of response inhibition and cognitive regulation, including Stroop, Trail Making Test, and verbal fluency paradigms [39, 41]. In addition, the Corsi Blocks results indicated a trend toward poorer visuospatial working memory in the SCA6 group, although without statistical significance, a finding consistent with evidence of working memory vulnerability in SCAs. However, tasks such as the Corsi Blocks also require precise upper-limb motor execution to reproduce the sequence, and motor imprecision may interfere with span performance independently of memory capacity, which may have contributed to the performance observed in this population [6, 42–44].

Regarding executive planning, performance on the Tower of London did not differ significantly between groups, suggesting relative preservation of this function in the present sample, a finding consistent with studies using analogous tasks [6].

Unlike previous findings, our study found significant verbal but not visual memory differences [43]. This discrepancy may reflect methodological and clinical variability among ataxias; however, our findings align with evidence of greater impairment in complex verbal recall, with preserved word recognition [42]. An unstable learning curve in 77% of our patients raises the question of whether delayed recall deficits reflect retrieval failure only or also impaired storage. These findings are consistent with prior evidence linking verbal memory and fluency impairments to frontocerebellar circuits connecting the cerebellum and prefrontal cortex [39]. The poorer performance on Rey Figure copy and recall is consistent with reports of subtle difficulties in visuospatial processing in cerebellar ataxias, although the contribution of visuomotor integration, graphomotor demands, and organizational planning to these results cannot be ruled out, and the observed differences may reflect an interaction between cognitive and motor/visuomotor factors rather than an isolated visuoconstructive impairment [41–43].

Our patients showed poorer performance on the Boston Naming Test, consistent with reports of subtle naming and fluency difficulties in SCA6 [42, 43]. Beyond cognitive alterations, patients with SCA6 also exhibited affective symptoms, with higher levels of anxiety. Depressive scores were higher in the SCA6 group compared with controls, although without statistical significance. This absence of significance may reflect, among other factors, variability across assessment instruments, as previous studies have reported depression prevalence ranging from 17% to nearly 50% depending on the instrument used [45–47].

The distribution of CCAS-S categories in this sample is consistent with the instrument’s intended use to detect cognitive-affective involvement in genetically confirmed SCA6, with the majority of patients classified in the definite category. However, the fact that all control participants were classified within the possible or probable categories suggests limited specificity at these lower thresholds, a pattern previously observed in other studies involving control groups and that has informed ongoing discussions about the need for refined diagnostic cutoffs. In this context, the definite category represents the most clinically robust descriptive finding, while between-group comparisons in the present study were based on the number of failed CCAS-S domains. Previous studies assessing SCA6 with neuropsychological batteries also identified poorer performance in this population, corroborating the findings of the present study [48, 49].

The correlations observed suggest that cerebellar dysfunction in SCA6 may be associated with poorer performance across multiple cognitive measures, particularly those involving visuoconstructive and visuomotor integration, naming, and time-dependent measures sensitive to executive control. The associations between the number of failed CCAS-S domains and scores on the Boston Naming Test, Block Design, estimated IQ, Stroop Test, and Rey Figure Copy are consistent with the possibility that these measures may capture aspects of non-motor cerebellar involvement. However, given that several of these measures involve motor, visuomotor, or speech output components, the observed associations may also partly reflect the impact of cerebellar motor dysfunction on task execution, and a purely cognitive interpretation of these correlations cannot be assumed. The positive correlation between SARA and number of failed CCAS-S domains suggests that progression of motor dysfunction may be associated with cognitive-affective impairment, although causal interpretations cannot be established from the available data.

The present study has limitations that warrant consideration. The small sample size, cross-sectional design, and absence of premorbid data restrict broader interpretations. Caution is warranted when interpreting self-report measures, which may not fully capture affective symptoms, and the long duration of testing sessions may have introduced fatigue effects. All statistical analyses should be regarded as exploratory; the absence of correction for multiple comparisons increases the risk of false-positive findings, and the small sample renders correlation estimates particularly sensitive to individual observations. Regarding language assessment, confrontation naming was evaluated using the Boston Naming Test and semantic and phonemic verbal fluency was analyzed as a subtask within the CCAS-S, only in terms of pass/fail per domain, without independent reporting of continuous scores, which limits the granularity of the linguistic analysis. Furthermore, social cognition was not included in the assessment battery. Recent studies have identified social cognition and detailed language assessment as relevant in cerebellar disorders, and their more comprehensive evaluation should be considered in future studies with larger samples, longitudinal designs, and multimodal approaches [50].

## Conclusion

The findings of the present exploratory study suggest that impairment in SCA6 may extend beyond the motor domain, affecting cognitive and affective functions essential for autonomy, including verbal episodic memory, performance on time-dependent measures sensitive to inhibitory control and selective attention, tasks involving visuoconstructive and visuomotor integration, and some aspects of language, alongside elevated levels of anxiety. These findings, combined with the subjective distress arising from perceived functional loss, support the relevance of broader clinical approaches integrating cognitive rehabilitation, psychotherapy, and occupational therapy alongside conventional treatments. Given the small sample size, the absence of correction for multiple comparisons, and the potential contribution of motor and processing-speed factors to performance on several measures, these results should be interpreted as preliminary. They highlight the relevance of standardized neuropsychological assessment protocols in this population and may inform more individualized therapeutic strategies, pending replication in larger, methodologically controlled samples.

## Data Availability

All data supporting the conclusions of this study are available in the article, presented in the analyses in terms of mean, standard error, and standard deviation.
